# A new precipitation and drought climatology based on weather patterns

**DOI:** 10.1002/joc.5199

**Published:** 2017-07-13

**Authors:** Douglas Richardson, Hayley J. Fowler, Christopher G. Kilsby, Robert Neal

**Affiliations:** ^1^ School of Civil Engineering and Geosciences Newcastle University Newcastle upon Tyne UK; ^2^ Department of Weather Science Met Office Exeter UK

**Keywords:** meteorological drought, precipitation, climatology, weather patterns, Lamb weather types, UK, standardized precipitation index, drought severity index

## Abstract

Weather‐pattern, or weather‐type, classifications are a valuable tool in many applications as they characterize the broad‐scale atmospheric circulation over a given region. This study analyses the aspects of regional UK precipitation and meteorological drought climatology with respect to a new set of objectively defined weather patterns. These new patterns are currently being used by the Met Office in several probabilistic forecasting applications driven by ensemble forecasting systems. Weather pattern definitions and daily occurrences are mapped to Lamb weather types (LWTs), and parallels between the two classifications are drawn. Daily precipitation distributions are associated with each weather pattern and LWT. Standardized precipitation index (SPI) and drought severity index (DSI) series are calculated for a range of aggregation periods and seasons. Monthly weather‐pattern frequency anomalies are calculated for SPI wet and dry periods and for the 5% most intense DSI‐based drought months. The new weather‐pattern definitions and daily occurrences largely agree with their respective LWTs, allowing comparison between the two classifications. There is also broad agreement between weather pattern and LWT changes in frequencies. The new data set is shown to be adequate for precipitation‐based analyses in the UK, although a smaller set of clustered weather patterns is not. Furthermore, intra‐pattern precipitation variability is lower in the new classification compared to the LWTs, which is an advantage in this context. Six of the new weather patterns are associated with drought over the entire UK, with several other patterns linked to regional drought. It is demonstrated that the new data set of weather patterns offers a new opportunity for classification‐based analyses in the UK.

## 1 Introduction

Weather pattern classifications (also called weather types, circulation types or circulation patterns) are useful for characterizing the broad‐scale atmospheric circulation over a given region and time‐scale (often daily; Huth *et al.,* 2008). They allow for general estimates of climatic variables such as wind direction and speed, temperature and precipitation. There are various different classifications of weather patterns. Two of the most well‐known European examples are the Grosswetterlagen (GWL; Hess and Brezowsky, 1952) and the Lamb weather types (LWTs; Lamb, 1972; Jenkinson and Collison, 1977). These classifications are intended to be used for many different applications, whereas some studies develop their own problem‐specific classification system (Bárdossy and Filiz, 2005; Philipp *et al.,* 2007; Casado *et al.,* 2009; Prein *et al.,* 2016). A comprehensive review of weather pattern classifications and applications was provided by Huth *et al*. (2008).

Most weather pattern studies focussed on aspects of UK precipitation climatology have used LWTs, as these are defined on a region centred over the British Isles. Wilby *et al*. (1994) generated daily precipitation time series for several sites in England by relating precipitation to LWTs. These data were then input to a hydrological model to simulate daily flows. Fowler and Kilsby (2002a) linked LWTs to the winter and summer North Atlantic Oscillation (NAO; Walker and Bliss, 1932) and in turn to precipitation in Yorkshire, England. Also for Yorkshire, Fowler *et al*. (2005) simulated climate change scenarios using a stochastic precipitation model conditioned on LWTs, whilst Malby *et al*. (2007) found associations between Lake District orographic precipitation variations and changes in wet circulation types. LWTs have also been studied in the context of flooding and heavy precipitation in the UK. Results from a hydro‐chemical model used by Wilby (1993) suggested flood frequencies in the East Midlands, England, are linked to changes in the occurrence of cyclonic and anticyclonic LWTs. Wilby (1998) successfully reproduced low‐frequency heavy daily precipitation incidence by relating such events to LWTs for several sites in central and southern England. However, the model did not capture variations in mean wet day probabilities or persistence, which the author attributes to general deficiencies of the LWT classification system and the method used to group individual weather types together. Of the 27 objective LWTs, Pattison and Lane (2012) found that just five accounted for over 80% of recorded extreme floods in Carlisle in northwest England. Burt and Ferranti (2012) showed that an increase in winter heavy precipitation in upland northern England was linked to an increase in the amount of precipitation associated with westerly LWTs. The same authors also showed that a decrease in summer heavy precipitation in the same region was linked to a decrease in the amount of precipitation associated with cyclonic LWTs.

Studies investigating the relationship between weather patterns and drought in the UK are rarer and normally do not consider the UK as a whole, instead focussing on a small number of regions. Wilby (1993) showed that the occurrence of drought in the East Midlands is related to the frequency of anticyclonic LWTs. Phillips and McGregor (1998) analysed frequencies of LWTs during droughts in southwest England, finding that drought distribution in the region depends on the position of the controlling anticyclone. Using a similar methodology, Fowler and Kilsby (2002b) demonstrated that Yorkshire droughts between 1881 and 1998 were typically associated with changes in frequencies of cyclonic and anticyclonic LWTs, with further differences in easterly and westerly type occurrences for subregional droughts. Away from LWTs, Fleig *et al*. (2011) related objective GWL (James, 2007) to a hydrological drought index in six regions covering Britain and Denmark. They found that of six weather patterns associated with drought, five feature a centre of high pressure to the north, although the governing pattern varied between regions and for drought events within each region.

A new set of weather patterns have been developed by the Met Office (Neal *et al.,* 2016). This classification consists of 30 weather patterns that are representative of the general atmospheric circulation over the UK and surrounding North Atlantic Ocean and European area, and will be referred to as MO‐30. MO‐30 has two main advantages over LWTs. First, the 30 patterns are derived objectively from first principles, without *a priori* categorization of the resultant flow over the UK. By contrast, LWTs are preordained categories of flow direction (i.e. northerly, south‐westerly, etc.) to which daily pressure patterns are (objectively or subjectively) assigned. Second, the patterns are defined over a much larger area than that used for the LWTs. By including much of the North Atlantic Ocean, MO‐30 better captures the large‐scale atmospheric systems that drive weather in Europe. Also, the larger region size means that it can be applied to other European regions, whereas LWTs must be redefined for studies outside the UK (see Lorenzo *et al.,* 2008, for an example in Spain). A further set of eight weather patterns, named MO‐8, has been defined by clustering types from MO‐30. This is intended for use in long‐range and seasonal forecasts.

MO‐30 and MO‐8 are used by the Met Office for several medium‐ to long‐range probabilistic forecasting applications. Ensemble member forecast scenarios are objectively assigned to the closest matching weather pattern definition, providing a probabilistic insight into the occurrence of different weather patterns throughout the forecast period. Once weather pattern characteristics are understood, in terms of their climatologies or impact, it then becomes possible to interpret forecast output and describe likely consequences. Two operational weather pattern forecast applications used at the Met Office are a tool for predicting air flow from Iceland that could potentially bring volcanic ash over UK airspace, and a tool for UK coastal flooding, with more applications under development.

There are three objectives to this study. First, an exploratory analysis of several features of the new classification, such as weather pattern frequencies and precipitation associated with each pattern. LWTs will be used as a comparison. Second, an investigation of how the new data set relates to the UK precipitation climatology, which on a monthly time‐scale will be defined by the standardized precipitation index (SPI; McKee *et al.,* 1993). Third, this study will expand upon previous work by linking weather patterns to drought in the UK as a whole, rather than on smaller regions. Drought will be quantified using the drought severity index (DSI) after Phillips and McGregor (1998) and Fowler and Kilsby (2002b). Whilst the example provided is for the UK, the methodology is transferrable to other regions within the domain of the weather patterns.

This study is organized as follows. Section 2 describes the data sets used. Methodology for associating weather patterns with daily precipitation and linking their monthly frequencies to drought indices is detailed in Section 3. Results are shown in Section 4, a discussion is in Section 5 and conclusions are presented in Section 6.

## 2 Data

**Figure 1:**
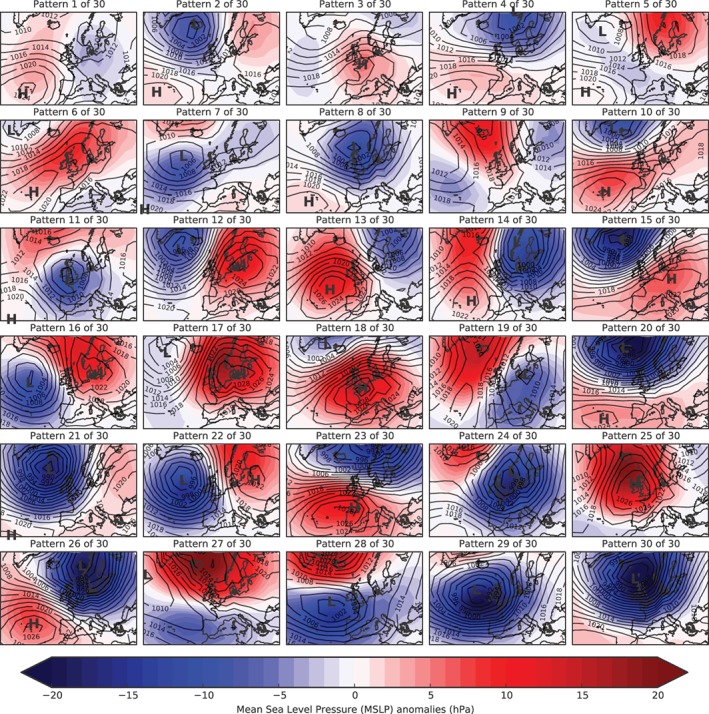
Definition of each weather pattern in MO‐30. Red shading is for positive mean sea level pressure (MSLP) anomalies (hPa) and blue shading is for negative MSLP anomalies. From Neal et al. (2016).

**Figure 2:**
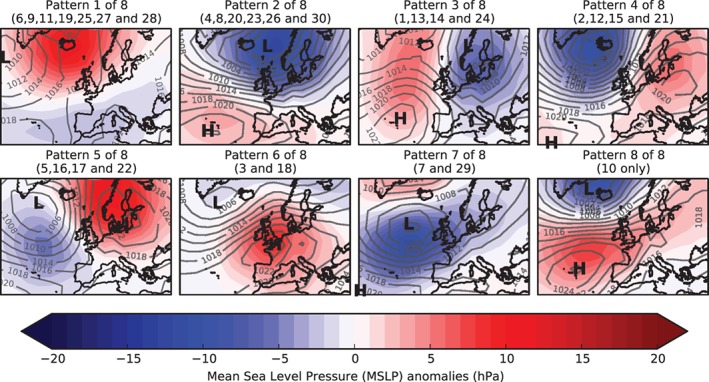
Definition of each weather pattern in MO‐8. Sub‐patterns from MO‐30 are listed in parentheses. Red shading is for positive mean sea level pressure (MSLP) anomalies (hPa) and blue shading is for negative MSLP anomalies. From Neal et al. (2016).

The methodology for deriving MO‐30 and MO‐8 is described in Neal *et al*. (2016). Briefly, 154 years (1850–2003) of daily mean sea level pressure (MSLP) fields from the European and North Atlantic Daily to multi‐decadal climate variability data set (EMULATE; Ansell *et al.,* 2006) were grouped into 30 distinct clusters using a simulated annealing technique (Philipp *et al.,* 2007; Huth *et al.,* 2008). The data have a spatial resolution of 5° latitude and longitude; the domain used in the clustering was 30°W–20°E; 35°–70°N, covering most of Europe and the North Atlantic. Daily historic weather pattern classifications are available from the EMULATE period (1850–2003), and have been extended from 2004 to the present using the European Centre for medium‐range weather forecasts ERA‐Interim data set (Dee *et al.,* 2011). Table 1 details a description of each weather pattern and Figure 1 gives their definition according to MSLP anomalies. MO‐8 was produced by repeatedly clustering patterns from MO‐30 according to spatial correlation between the pairs of patterns. As a result, each pattern in the smaller set comprises between one and seven patterns from the larger set. Table 2 contains descriptions of each pattern in MO‐8 and Figure 2 shows the MSLP anomaly maps.

**Table 1:** For each weather pattern in MO‐30, a description of the resultant flow over the UK, the historic occurrence (%) between 1850 and 2015 for all months (A), the winter half‐year (W) and the summer half‐year (S) and objectively assigned LWT class is listed.

Weather pattern	Flow description	Historic occurrence (%)			LWT
		**A**	**W**	**S**	
1	Neutral north‐westerly	6.45	2.28	10.60	U
2	Cyclonic south‐westerly	5.63	3.16	8.09	SW
3	Anticyclonic south‐westerly with a ridge over northern France	5.26	2.59	7.92	ASW
4	Neutral westerly	4.89	2.99	6.78	W
5	Neutral southerly with a centre of high pressure over Scandinavia	4.86	2.85	6.86	S
6	Anticyclonic with a high pressure centre over the Azores	4.88	3.22	6.53	A
7	Cyclonic south‐westerly with a centre of low pressure west‐north‐west of Ireland	4.92	2.70	7.13	U
8	Cyclonic westerly with a low pressure centre near the Shetland Islands	4.62	3.20	6.04	C
9	Anticyclonic with high pressure over Iceland	4.48	2.91	6.05	A
10	Anticyclonic westerly with high pressure over the Azores	4.30	3.43	5.17	W
11	Cyclonic with a low pressure centre over southern Britain	3.65	2.83	4.47	C
12	Anticyclonic south–south‐westerly with high pressure over Poland	3.60	4.23	2.97	SW
13	Anticyclonic north‐westerly with high pressure south‐west of Ireland	3.49	4.03	2.95	NW
14	Cyclonic north–north‐westerly with low pressure centred over southern Sweden	3.07	3.87	2.27	NW
15	Neutral south‐westerly. Very windy for northwest Britain	3.02	4.39	1.65	SW
16	Anticyclonic south–south‐easterly with high pressure near Denmark	2.72	3.19	2.24	S
17	Anticyclonic east‐south‐easterly with a high pressure centre over Denmark	2.57	3.99	1.16	AS
18	Anticyclonic south‐westerly with high pressure over northern France	2.59	4.38	0.81	ASW
19	Neutral northerly	2.55	3.83	1.28	N
20	Cyclonic westerly with a deep centre of low pressure near Iceland. Very windy	2.57	3.98	1.17	W
21	Cyclonic south‐westerly with a deep low south of Iceland. Strong winds	2.52	3.57	1.46	SW
22	Cyclonic southerly with low pressure west of Ireland	2.28	3.15	1.42	S
23	Neutral westerly with high pressure north of Spain. Windy	2.36	3.90	0.82	W
24	Cyclonic northerly with low pressure centred over the North Sea	1.98	3.08	0.89	C
25	Anticyclonic northerly with high pressure over Northern Ireland	2.08	3.37	0.80	A
26	Cyclonic north‐westerly with low pressure centred near Norway. Very windy	1.95	3.19	0.71	NW
27	Anticyclonic easterly with high pressure over the Norwegian Sea	1.82	3.29	0.35	SE
28	Cyclonic south‐easterly with low pressure southeast of the UK	1.72	3.00	0.45	CSE
29	Cyclonic south–south‐westerly with a deep low centre of pressure west of Ireland. Very windy	1.64	2.73	0.55	C
30	Cyclonic west‐north‐westerly with deep low pressure southeast of Iceland. Very windy	1.52	2.64	0.41	CW

**Table 2:** As for Table 1, but for MO‐8. Includes column detailing the patterns from MO‐30 clustered into each MO‐8 pattern.

Weather pattern	Sub‐patterns from MO‐30	Flow description	Historic occurrence (%)			LWT
			A	W	S	
1	6, 9, 11, 19, 25, 27 and 28	Blocked, negative NAO pattern	21.19	22.46	19.92	U
2	4, 8, 20, 23, 26 and 30	Zonal, positive NAO pattern	17.92	19.91	15.93	W
3	1, 13, 14 and 24	Neutral north‐westerly with low pressure northeast of the UK and high pressure to the southwest	14.99	13.26	16.71	NW
4	2, 12, 15 and 21	Cyclonic south‐westerly with low pressure centred near Iceland	14.76	15.35	14.17	SW
5	5, 16, 17 and 22	Anticyclonic southerly with high pressure near Denmark and low pressure southwest of the UK	12.43	13.18	11.69	S
6	3 and 18	Anticyclonic west‐south‐westerly with a centre of high pressure over northern France	7.85	6.97	8.73	ASW
7	7 and 29	Cyclonic south‐westerly with low pressure west of Ireland	6.56	5.44	7.67	CSW
8	10	Anticyclonic westerly with high pressure over the Azores	4.30	3.43	5.17	W

**Figure 3:**
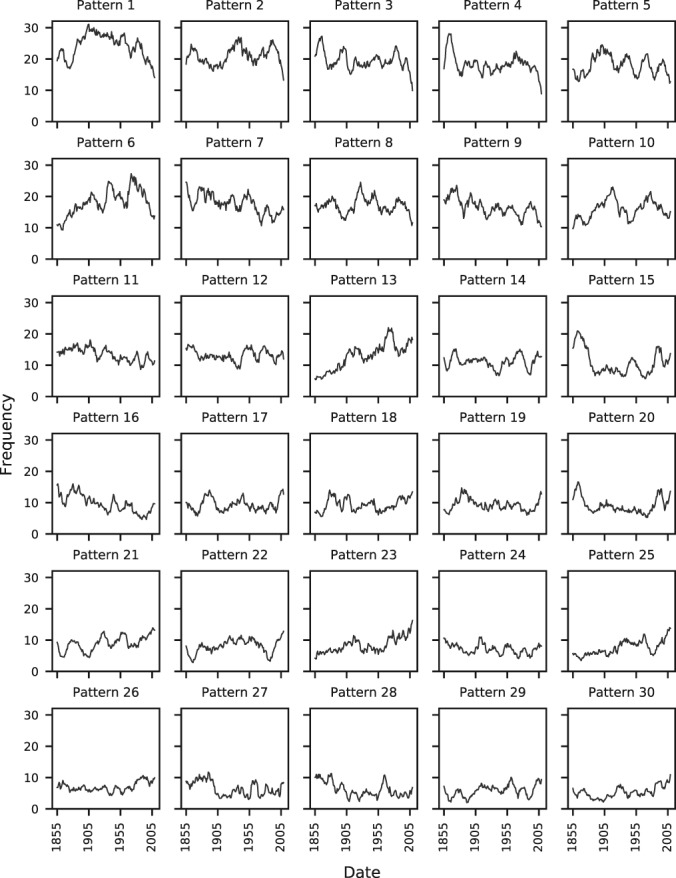
Eleven‐year moving average frequencies of each weather pattern in MO‐30. Dates represent the central year of each 11‐year window.

Individual patterns will be referred to as WP*i*, with *i* indicating the pattern number. Neal *et al*. (2016) ordered the patterns in both sets according to their annual historic occurrence in the period used for the clustering technique (1850 to 2003), with WP1 occurring most often and the last pattern (WP30 or WP8) least often. The use of only MSLP anomalies in the clustering results in some seasonal grouping of patterns. This is particularly true for MO‐30. Table 1 shows that lower‐numbered patterns occur more often during the summer half‐year (April through September; weak MSLP anomalies) and higher‐numbered patterns occur more in winter (October through March; strong MSLP anomalies). Figure 3 shows the 11‐year moving average frequency of each pattern in MO‐30. There is strong interannual variability for most patterns, and suggestions of a trend in several. Further discussion of weather pattern frequencies and a comparison with long‐term trends in LWT occurrences is in Section 5.

We objectively classify each weather pattern as a LWT following the method in Jones et al. (1993), see Table 1 and Table 2. Using 16 grid points in the domain 20°W–10°E; 45°–65°N, values of wind flow and vorticity are calculated from daily MSLP data. LWTs are then defined using a set of rules related to the relative strength of these values. LWTs can be one of eight flow direction types (N, NE, E, SE, S, SW, W and NW) and/or one of anticyclonic (A) and cyclonic (C). Flow directions may be combined with cyclonic/anticyclonic types where appropriate. There is an additional ‘unclassified’ LWT to represent light indeterminate flow, denoted U; giving a total of 27 LWTs. Table 3 details the number of patterns from MO‐30 assigned to each LWT. We discuss this classification and draw comparisons with LWT frequencies in Section 5. The LWT series between 1871 and 2015, derived using reanalysis products, is from Jones et al. (2013b). Historic occurrence of each LWT in this period is in Table 3.

**Table 3:** LWT historic occurrence (%) between 1871 and 2015 and the number of weather patterns from MO‐30 assigned to each LWT by the objective classification method.

LWT	Historic occurrence (%)	No. of patterns from MO‐30
A	20.58	3
C	13.94	4
SW	9.45	4
W	9.02	4
S	5.64	3
NW	4.97	3
N	3.29	1
AW	3.20	0
SE	3.16	1
ASW	2.73	2
CSW	2.49	0
CW	2.24	1
CS	1.97	0
E	1.84	0
ANW	1.82	0
NE	1.74	0
AS	1.65	1
CNW	1.62	0
AN	1.19	0
ASE	1.17	0
CN	1.14	0
U	1.06	2
CSE	1.02	1
AE	1.00	0
ANE	0.80	0
CNE	0.67	0
CE	0.62	0

**Figure 4:**
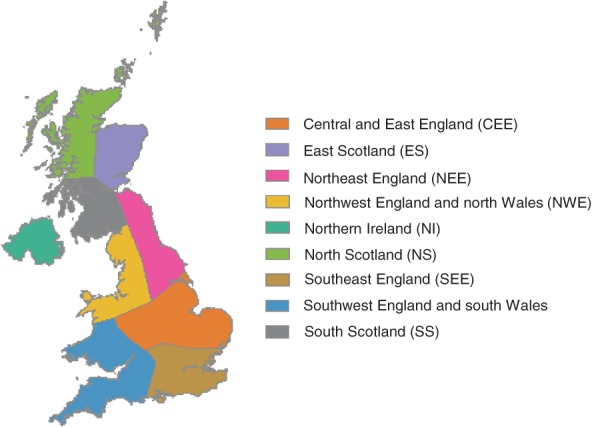
Regional boundaries of the precipitation data set.

UK daily and monthly precipitation data are from the Met Office Hadley Centre UK Precipitation data set (Alexander and Jones, 2000). The UK is split into nine regions (Figure 4) originally defined by Wigley et al. (1984): northeast England (NEE), central and east England (CEE), southeast England (SEE), southwest England and south Wales (SWE), northwest England and north Wales (NWE), east Scotland (ES), southwest and south Scotland (SS), northwest and north Scotland (NS) and Northern Ireland (NI). Regional precipitation series are available as daily totals from 1931 to the present and monthly totals from 1873 (for NEE, CEE, SEE, SWE and NWE) or 1931 (for the remaining regions). The data are estimates of the regional average calculated using at least seven evenly distributed precipitation gauging stations, see Alexander and Jones (2000) for details.

## 3 Methodology

### 3.1 Associating daily precipitation with weather patterns and LWTs

**Figure 5:**
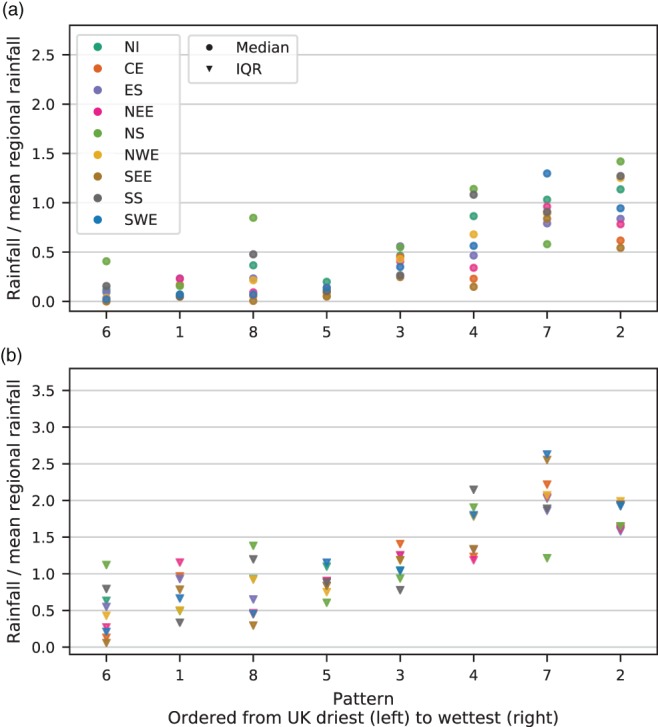
(a) Median and (b) interquartile range (IQR) of daily rainfall 1931–2015 for each region and each pattern in MO‐8, expressed as the proportion of rainfall relative to the regional average. Patterns are ordered left to right from the lowest UK mean rainfall (i.e. averaged across all regions) to the highest.

**Figure 6:**
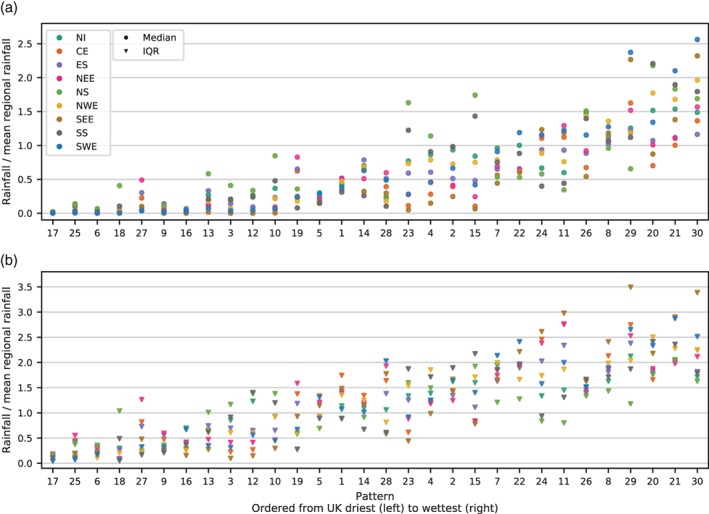
As Figure 5 but for each pattern in MO‐30.

Precipitation data between 1931 and 2015 are associated by weather pattern, giving a distribution of daily precipitation totals for each pattern. To allow comparison between regions, these data are divided by the regional mean daily precipitation. The result is a precipitation distribution for each weather pattern expressed as a proportion of the regional average. The median and interquartile range (IQR) for each of these distributions are displayed in Figure 5 for MO‐8 and Figure 6 for MO‐30. Ideally, a weather pattern classification would show distinct distributions for each pattern. From Figure 5, it is clear that this is not the case for MO‐8. Across all regions, half of the weather patterns (WP1, WP5, WP6 and WP8) have very similar median and IQR; WP2 and WP7 are also very similar. This is because the clustering process used to derive the patterns does not use precipitation but instead spatial correlation of MSLP anomalies. As a result, several patterns from MO‐8 are composed of patterns from MO‐30 that show very different precipitation distributions. For example, Table 2 shows that WP1 (a negative NAO‐like pattern) contains a mixture of patterns featuring cyclonic and anticyclonic characteristics. Figure 6 shows that the patterns in MO‐30 feature more distinct precipitation distributions, although there remain several subsets that appear similar (e.g. WP13 and WP3 or WP4 and WP2). The precipitation distributions of each pattern in MO‐8 are considered too similar to warrant further analysis in the context of precipitation climatology, so this set is excluded from the remainder of the study.

**Figure 7:**
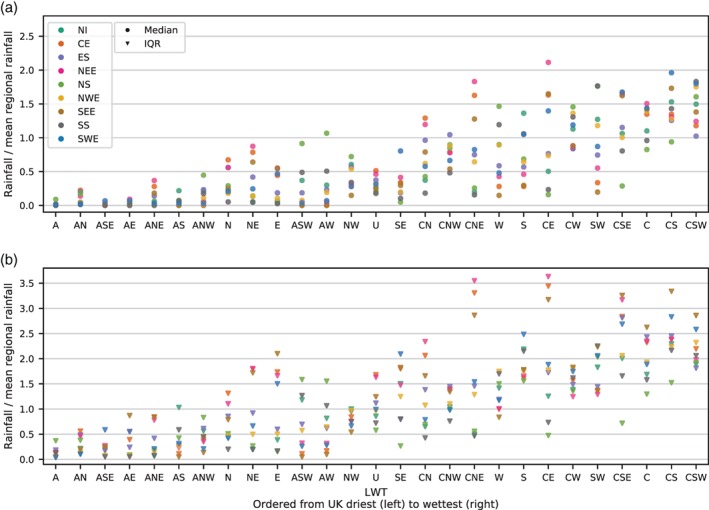
As Figure 5 but for LWTs.

The same method is applied to the LWTs between 1871 and 2015, with median and IQR of daily rainfall associated with each LWT shown in Figure 7. For many of the wet LWTs, the IQR is much higher than for wet patterns in MO‐30. In particular, the daily rainfall variability associated with cyclonic easterly LWTs (CE, CNE and CSE) in eastern England is large. The lower variability of rainfall associated with patterns in MO‐30 compared to with LWTs suggests the former is better suited to rainfall analyses in the UK.

### 3.2 Linking MO‐30 frequencies with the SPI

We now analyse UK monthly precipitation climatology in relation to MO‐30, using the SPI. Developed originally for drought applications, the SPI is equally valid for wet periods. The SPI is calculated by fitting a parametric probability density function to precipitation data and transforming it to the standard normal distribution (see Lloyd‐Hughes and Saunders (2002) for full details). Standardization means the SPI is comparable across different regions. Negative (positive) SPI values signify the degree of dryness (wetness). Typically, monthly precipitation data used in SPI calculation is first aggregated over some time‐scale in months, *k*, resulting in an SPI series that represents the degree of dryness/wetness over a chosen period, denoted SPI‐*k*. For example, SPI‐3 might be used to describe meteorological or agricultural drought conditions, whilst SPI‐12 is more suitable for describing long‐term hydrological drought (McKee *et al.,* 1993, 1995). There is ongoing debate about which statistical distribution should be fitted to monthly precipitation data. McKee *et al*. (1993) originally used the gamma distribution; the Pearson type III (Guttman, 1999) and Weibull (Sienz *et al.,* 2012) have also been suggested. The choice of distribution is important, as it has a significant effect on the resultant SPI values (Sienz *et al.,* 2012; Guerreiro *et al.,* 2017).

SPI series for k = 3, 6 and 12 are calculated for each region. To maintain comparability across regions, the distributions are fitted using the common period, 1931 to 2015. The distributional fit of seven parametric distributions (the gamma, Weibull, exponential, Pearson type III, generalized extreme value, Gumbel and normal distributions) is assessed on each precipitation series using a modified mean square error (MSE) metric defined by Papalexiou *et al*. (2013) and used for SPI distribution fitting by Guerreiro *et al*. (2017). This modified metric is advantageous compared to the classical MSE because it gives as much weight to values in the tails of the distribution as those in the middle. This is important for assessing extreme values. The modified MSE is defined as:

$$\mathrm{MSE}=\frac{1}{N}\sum_{i=1}^{N}{\left(\frac{F\left(x_{i}\right)}{F_{N}\left(x_{i}\right)}-1\right)}^{2},$$

where, *N* is the sample size, *F*(*x*) is the probability density function given any theoretical distribution and *F*_*N*_(*x*_*i*_) is the probability of exceeding *x*_*i*_ using the empirical distribution

$$F_{N}\left(x_{i}\right)=\frac{r\left(x_{i}\right)}{N+1},$$

where, *r*(*x*_*i*_) is the rank of *x*_*i*_. The best‐fitting distribution is that which yields the lowest MSE. For each aggregated precipitation series, the MSE is calculated monthly and then summed to give an estimate of the annual MSE. The lowest annual MSE scores for the majority of the series are obtained by fitting the Pearson type III distribution, with the gamma and Weibull distributions also represented. The chosen (series‐specific) distributions are fitted to the precipitation series using maximum likelihood estimation and transformed to the standard normal distribution, yielding the SPI series.

The daily weather pattern series is aggregated to monthly frequencies for each pattern in MO‐30. By examining pattern frequencies during anomalously dry or wet periods it is possible to ascertain which are associated with these conditions. Here, “dry” and “wet” periods are defined as months where SPI‐*k* ≤  − 1 and SPI‐*k* ≥ 1, respectively, corresponding to roughly 16% probability at each tail of the distribution. As SPI values are calculated based on precipitation aggregated over *k* months, we calculate weather pattern frequencies over the same time‐scales. Therefore, *k* = 3 , 6 and 12 monthly summed frequencies are calculated for each weather pattern. The mean of these *k*‐monthly frequencies during dry/wet periods is divided by the mean of the *k*‐monthly frequencies over the entire record, giving the frequency anomalies of each weather pattern during dry/wet periods. This is then expressed as a percentage anomaly (*PA*). For a given weather pattern and time‐scale, *PA* is defined as:

$$\mathrm{PA}=100\left(\frac{\bar{y}}{\bar{x}}-1\right),$$

where, $\bar{x}$ is the mean frequency of the weather pattern over the whole record and $\bar{y}$ is the mean frequency of the weather pattern during dry/wet periods. For *PA* = 0, the average frequency of a particular weather pattern during dry/wet periods is the same as the average over the entire record. A negative *PA* implies the weather pattern occurs less frequently than normal, and vice versa for a positive *PA*. To enhance the readability of this article, *PA*s are referred to simply as “anomalies”. The significance of the anomalies are assessed using Welch's *t‐*test (Welch, 1947), with the null hypothesis that the means of the two samples (frequencies during dry/wet periods and frequencies over the remainder of the record) are the same. An assumption of this test is that the data follow a normal distribution. However, as the frequencies are count data exhibiting heavily skewed behaviour, we must first assess its suitability by checking whether nominal significance is preserved. This is done by repeatedly resampling the frequency data and performing the test on each sample with significance level *α* = 0.05. The type I error rate (incorrect rejection of a true null hypothesis) is only slightly inflated above nominal significance (typically around 0.058) in a few cases; this is deemed adequate for the purpose of the study. The method described in this section is used for the winter and summer half‐years (October through March and April through September, respectively) as well as annually.

For brevity, only selected results are presented. An anomaly for the dry/wet criterion is typically complemented by an anomaly of the opposite sign and roughly equal magnitude for the other criterion. As we focus on droughts, SPI anomalies are presented for dry periods, with the corresponding wet period anomalies shown in the Supporting Information. Furthermore, results are not presented for SPI‐6 and SPI‐12. This is because they are very similar to SPI‐3 results, although the anomaly magnitudes typically reduce as *k* increases. This is logical, as by extending the period over which monthly frequencies are considered, the likelihood of a larger range of weather patterns occurring increases. That is, a longer‐term dry (wet) spell is more likely to contain a greater number of wet (dry) weather pattern occurrences than for a shorter‐term spell. A further reason for omitting SPI‐6 and SPI‐12 results is that, as winter and summer are considered as half‐years, the weather pattern frequencies for SPI‐6 and SPI‐12 include information from the preceding season. This is generally not the case for SPI‐3, with much of the averaging taking place only in the considered season.

### 3.3 Linking MO‐30 frequencies with the DSI

For drought‐specific applications, the DSI is sometimes used. Based on cumulative precipitation deficits, rather than the *k*‐monthly “snapshot” nature of the SPI, it is more appropriate for analysing the evolution and accumulated intensity of a drought. Furthermore, the DSI is non‐parametric and does not suffer from the SPI's requirement of finding a suitable distribution to fit to the underlying data. Whilst the DSI is preferred here for drought analysis, the SPI is used in the previous section to assess wet, as well as dry periods, in which context it is more appropriate than the DSI. As with the SPI, the DSI can be calculated for different time‐scales and is denoted DSI‐*k*. The DSI calculation procedure is as follows:

1. For some time‐scale, *k*, let the precipitation anomaly in month *t* be *X*_*t*_. If *X*_*t*_ < 0 (i.e. precipitation is below the mean), and precipitation in the *k*‐monthly period *X*_*t*_ , *X*_*t* − 1_ ,  …  , *X*_*t* − *k* + 1_ is also below its *k*‐monthly mean then initiate a drought sequence in month *t* and set DSI‐*k*_*t*_ =  − *X*_*t*_.
2. For the next month, *t* + 1, the precipitation anomaly is *X*_*t* + 1_. Then DSI‐*k*_*t* + 1_ =  − *X*_*t* + 1_+ DSI‐*k*_*t*_, if and only if the *k*‐monthly mean of *X*_*t* + 1_ , *X*_*t*_ ,  …  , *X*_*t* − *k* + 2_ is not exceeded. If the *k*‐monthly mean is exceeded then DSI‐*k*_*t* + 1_ is set to zero and the drought is terminated. This step is repeated for the entire precipitation series.
3. The DSI‐*k* series is standardized by dividing the absolute deficit (in mm) by the mean annual precipitation and multiplying by 100. The index then expresses the cumulative precipitation deficit as a percentage of mean annual precipitation.

Note that the DSI can be negative when a precipitation surplus occurs over a time‐scale shorter than *k*. The DSI has been used for assessing historical and projected drought conditions in the UK (Phillips and McGregor, 1998; Fowler and Kilsby, 2002b; Blenkinsop and Fowler, 2007a; Rahiz and New, 2012, Rahiz and New, 2013; Rahiz and New, 2014), Iberia (Guerreiro *et al*., 2017) and Europe‐wide (Blenkinsop and Fowler, 2007b).

For each region, DSI series are calculated using the same reference period as for the SPI (1931 to 2015). For each series, a threshold is selected such that roughly 5% of values are above the threshold. The months that these values correspond to are named “drought months”. Some previous studies using DSI have selected arbitrary thresholds and defined a drought as when DSI exceeds this threshold over multiple locations (Phillips and McGregor, 1998; Fowler and Kilsby, 2002b). The regions used in this study, however, are large enough for a drought to occur in one region but not any other, justifying the use of different thresholds for different regions. As for SPI dry and wet periods, weather pattern *PA*s are calculated for drought months defined by the DSI series. However, Welch's *t*‐test is not suitable in this case as nominal significance is not preserved. This is probably due to the sample size of drought months being far smaller than that of SPI wet/dry periods, leading to the test's assumptions being more easily violated. Other tests, such as the Mann–Whitney *U*‐test (Mann and Whitney, 1947) and permutation *t*‐test were tried with similar results. Therefore, DSI *PA*s are reported without testing for statistical significance. As before, we examine annual, winter and summer seasons. Results are presented for DSI‐3, DSI‐6 and DSI‐12 to enable comparison of anomalies between droughts of different lengths. Winter and summer results are not shown, as they are qualitatively the same as for annual, particularly for DSI‐6 and DSI‐12.

## 4 Results

### 4.1 MO‐30 frequency anomalies during SPI wet and dry periods

#### 4.1.1 *Annual*

**Figure 8:**
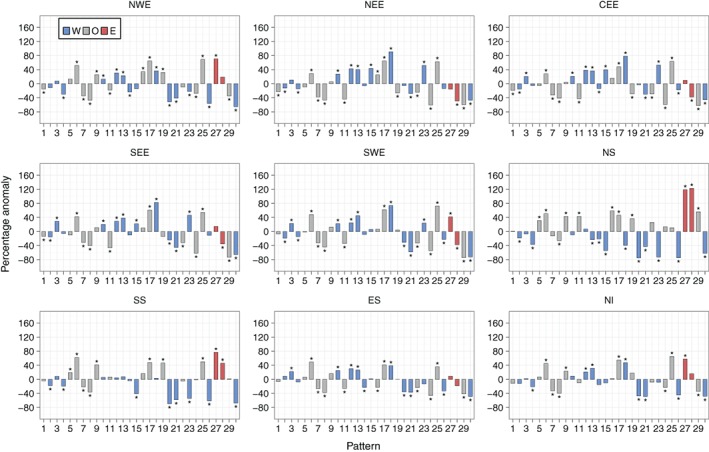
Annual (i.e. all months) three monthly mean frequency percentage anomalies of each weather pattern in MO‐30 during dry periods defined by SPI‐3 ≤ −1. Blue and red bars indicate that the weather pattern contains a westerly (W) or easterly (E) component in its LWT equivalent, respectively. Grey bars represent all other types (O). An asterisk indicates statistical significance at the 95% level.

Figure 8 shows the anomalies of three‐monthly mean frequencies during annual SPI‐3 dry periods for each region. The two weather patterns that occur statistically significantly more frequently during dry periods compared to normal across all regions are both anticyclonic LWT variants – WP6 and WP17. For wet periods, Figure S1 shows that three cyclonic or westerly variants (WP8, WP21 and WP30) occur significantly more frequently than normal for all regions. Differences in anomalies between eastern and western regions are apparent. From Figure 8, western regions generally see a larger increase than eastern regions in the occurrence of WP9 and WP27 during dry periods compared to normal. WP27 is a south‐easterly LWT so any precipitation associated with this pattern would mostly fall on East Britain. WP9 is anticyclonic, although it is hard to discern from Figure 1 why it is linked with more precipitation in western regions as there is no indication of flow direction. Dry periods in most eastern (northern and western) regions are associated with an increase (decrease) in the frequency of two patterns that would bring strong westerly winds over northern Britain – WP15 and WP23. From Figure S1, the opposite effect of these regional differences is apparent for wet SPI periods. Furthermore, western regions are associated with larger decreases in the occurrence of three windy, westerly patterns (WP20, WP21 and WP26) during dry periods than eastern regions (Figure 8). Dry periods in NEE, CEE, SEE and SWE are associated with a statistically significant decrease in the occurrence of WP28, with significant increases for NS and SS.

Results in NS differ to the other regions (Figure 8). In general, the anomalies are greater in magnitude. Also, many patterns that occur more frequently than normal during dry/wet periods in NS occur less often than the average for other regions, and vice versa. For example, the frequency of WP29 (a cyclonic LWT) is above average for NS yet is below average for all other regions except SS (for which it is near normal). The MSLP anomaly definition in Figure 1 implies strong south‐south‐westerly winds over the UK, so precipitation brought over the UK would fall heavily on other regions first, become moderate over SS before turning dry in NS. The differences between NS and other regions may partly be due to its location on the northern tip of the UK. It is exposed to both western and eastern coastlines, and so is on the front line of north‐westerly, northerly and north‐easterly winds.

#### 4.1.2 *Winter and summer*

Neal et al. (2016) suggest that knowledge of the seasonal behaviour of the weather patterns could give an indication of an extreme weather event. Higher‐numbered patterns occur more in winter than summer, so the occurrence of, say, WP30 in summer may indicate an extreme event on that day. This idea is extended here to monthly data by considering PAs calculated with respect to the seasonal average for winter and summer half‐years.

**Figure 9:**
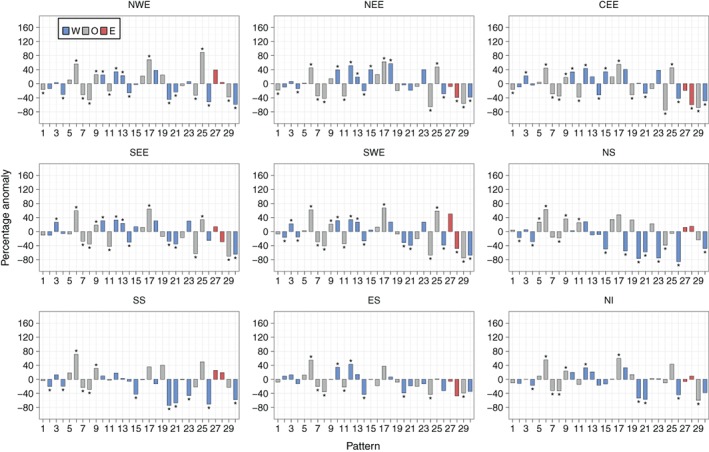
As Figure 8 but for summer.

**Figure 10:**
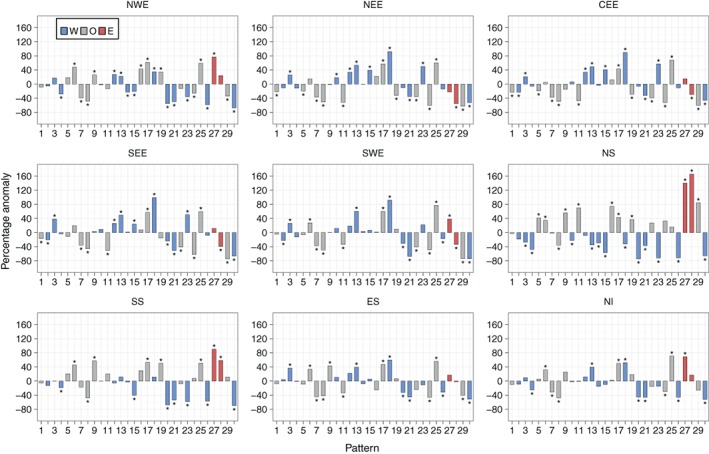
As Figure 8 but for winter.

Recall from Table 1 that lower‐ (higher‐) numbered patterns are more associated with summer (winter). Figure 9 shows that for some regions (particularly those in Scotland, but not NWE), summer SPI‐3 dry periods occur as a result of a decrease in the frequency of wet patterns that are associated with winter (e.g. WP20, WP21 and WP30), together with an increase in the occurrence of dry patterns that are associated with summer (particularly WP6). Winter SPI‐3 dry periods generally see larger changes in the occurrence of patterns that are associated with winter compared to the patterns that are associated with summer. This is shown in Figure 10, with higher‐numbered patterns tending to have higher‐magnitude anomalies than lower‐numbered patterns (except in ES). Wet SPI‐3 periods in summer are characterized by an increase in the occurrence of wintery, wet patterns more than a decrease in the occurrence of dry patterns (Figure S2). Figure S3 shows that for wet periods in winter, a greater number of weather patterns feature strong anomalies than in summer. For example, in NWE, six patterns show non‐significant anomalies in winter (Figure S2) compared to 11 patterns in summer (Figure S3).

### 4.2 Defining drought months and MO‐30 frequency anomalies

#### 4.2.1 *Identifying drought periods using DSI*

**Figure 11:**
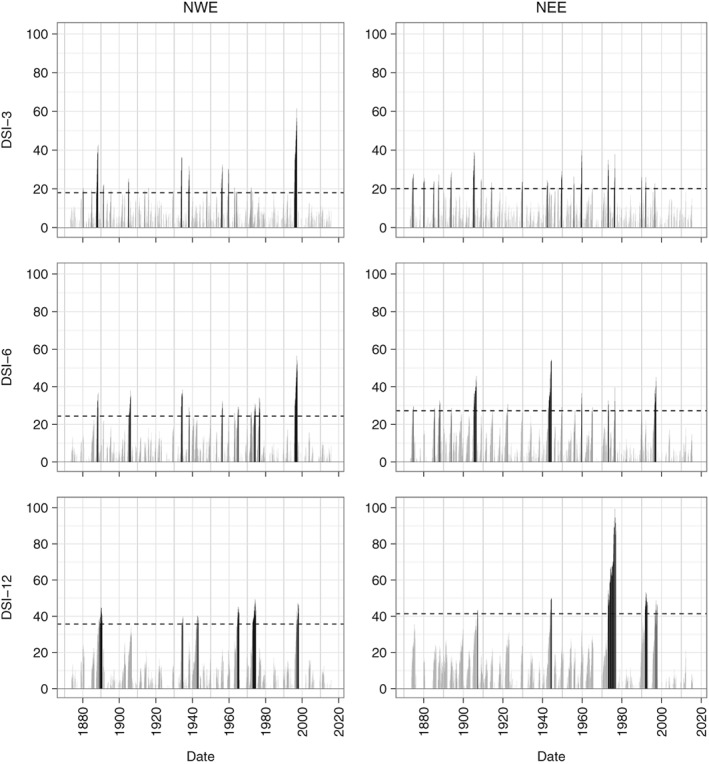
DSI series for NWE (left column) and NEE (right column) 1883–2015 indicated by grey bars. First row is DSI‐3, second row is DSI‐6 and third row is DSI‐12. Drought months are represented by the black bars. The dashed horizontal line indicates the threshold for which DSI values are considered drought months.

Figure 11 displays the DSI‐3, DSI‐6 and DSI‐12 series for NWE and NEE. As the time‐scale increases, the threshold increases and drought months (black bars in Figure 11) become clustered together; droughts become more intense, less frequent and longer in duration. This is consistent with results from other studies (Phillips and McGregor, 1998; Fowler and Kilsby, 2002b). Results for other regions feature the same behaviour (not shown). Notable droughts can be identified from Figure 11. The 1995–1996 drought is clearly visible in both regions. In NWE, for DSI‐3 and DSI‐6 this is the most intense drought in the record from 1873. Although still a major drought in the DSI‐12 series, it is matched in intensity by several other episodes such as the 1975–1976 drought and part of the long drought between 1890 and 1910 (see Marsh *et al*., 2007). For DSI‐3 in NEE there are many separate drought episodes of relatively low intensity. Other regions also feature a high frequency of droughts for this time‐scale, although they are generally less intense for northern and eastern regions. A notable feature of Figure 11 is how long and intense the mid‐1970s drought was for NEE when considering DSI‐12 (right column, third row). The DSI‐12 value of almost 100 is unmatched in any other region or at any other time‐scale and this drought accounts for the majority of the DSI values above the threshold (for this region and time‐scale). The more recent 2010–2012 drought is not evident in NWE or NEE but is for CEE, SEE and SWE regions (not shown). This is consistent with the exaggerated northwest‐southeast precipitation gradient observed during this period (Kendon *et al*., 2013).

#### 4.2.2 *MO‐30 frequency anomalies during drought months*

**Figure 12:**
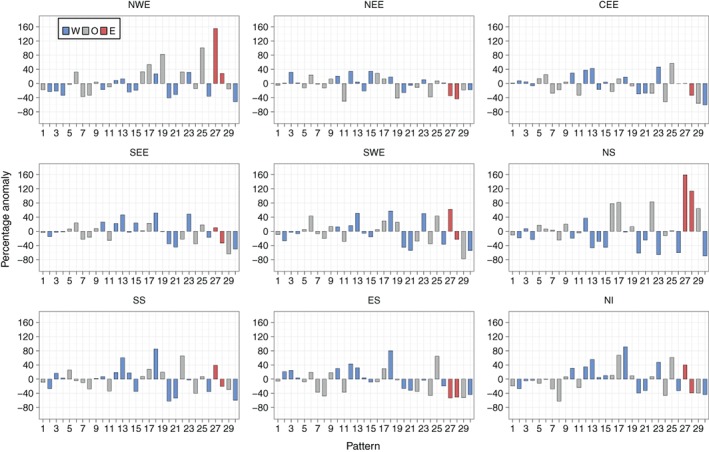
Annual (i.e. all months) three monthly mean frequency percentage anomalies of each weather pattern in MO‐30 during drought months defined by DSI‐3. Blue and red bars indicate that the weather pattern contains a westerly (W) or easterly (E) component in its LWT equivalent, respectively. Grey bars represent all other types (O).

Figure 12 shows the results for annual three‐monthly mean frequency anomalies for each region. Weather patterns that are associated with dry (wet) conditions defined by SPI typically occur more (less) frequently during drought months than normal. DSI drought months represent extreme dryness better than SPI dry periods, as they account for 5% of each series, compared to 16% for SPI. The smaller‐magnitude anomalies of lower‐numbered patterns (e.g. WP6 through WP9 in Figure 12) during drought months compared to SPI wet/dry periods (Figure 8) implies that greater‐intensity droughts are characterized by an increase or decrease in frequency of those weather patterns that occur less often annually (i.e. the higher‐numbered patterns).

The results for WP17 (an AS LWT) are surprising. It has the lowest UK mean daily precipitation between 1931 and 2015 (Figure 6) and occurs significantly more frequently during SPI‐3 dry periods than normal for all regions (Figure 8). However, it only occurs far more often than normal during DSI‐3 drought months for NWE, NS and NI (Figure 12). The regional differences in anomalies for DSI‐3 (Figure 12) correspond to those for SPI‐3 wet and dry periods. WP27 occurs more (less) frequently than normal during drought months in western (eastern) regions. This pattern is particularly associated with droughts in NWE and NS, occurring around 160% more than normal. WP15 and WP23 (westerly variants) are not as strongly associated with DSI drought months as they are for SPI‐3 dry periods in eastern regions. This is perhaps because neither pattern is dominated by anticyclonic conditions; both would bring strong winds over the UK. Drought in NWE, NS and NI is characterized by larger increases in the occurrence of dry patterns rather than decreases in the occurrence of wet patterns. The opposite is true for NEE and SEE, where droughts appear more associated with a decrease in wet weather types (e.g. WP29 and WP30 for SEE and WP11 for NEE).

**Figure 13:**
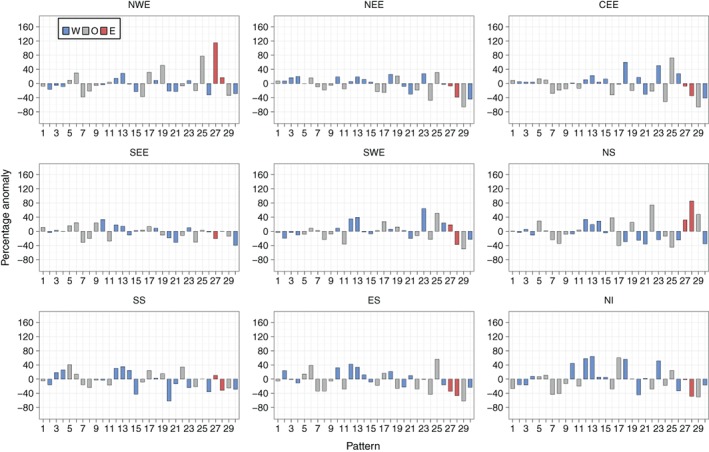
As Figure 12 but for DSI‐6.

**Figure 14:**
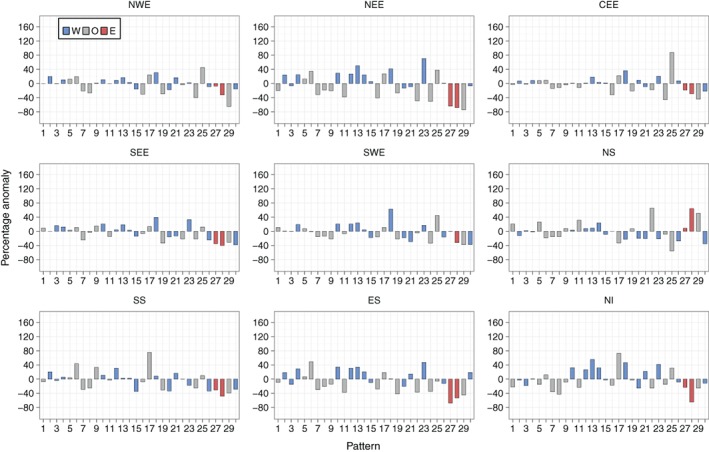
As Figure 12 but for DSI‐12.

Unlike SPI, increasing the time‐scale for DSI does not always yield lower‐magnitude anomalies. NEE displays some of the lowest magnitude anomalies for DSI‐3 (Figure 12), with similar results for DSI‐6 (Figure 13). When considering 12‐month droughts, however, anomaly magnitudes for this region increase to some of the greatest of all regions (Figure 14). Conversely, NWE features some of the largest positive anomalies for DSI‐3 (Figure 12) and DSI‐6 (Figure 13) compared to other regions, with droughts characterized by strong increases in the occurrence of WP19, WP25 and WP27. For DSI‐12, however, it is less clear which weather patterns cause drought in NWE, except perhaps a strong decrease in the occurrence of WP29 (Figure 14). Also of note is how, in some regions, DSI‐12 droughts are characterized by a smaller set of weather patterns than drought at smaller time‐scales. For example, in CEE, Figures 12 and 13 show DSI‐3 and DSI‐6 droughts are attributed to increases and decreases in a number of patterns' frequencies. By contrast, Figure 14 indicates DSI‐12 droughts are typified by a strong increase in the occurrence of WP25, followed by the decrease in occurrence of WP24 and WP29.

## 5 Discussion

### 5.1 MO‐30 and LWT frequencies

A significant feature of MO‐30 frequencies (Figure 3) is that, over the last 30 years of the record, days featuring stronger MSLP anomalies (WP12 through WP30 days) have become more frequent at the expense of days where MSLP anomalies are weaker (WP1 through WP11 days). These two groups of patterns are split seasonally, with WP1 through WP11 occurring more often during summer than winter and vice versa for the remaining patterns. This increase in the amount of “wintery” days featuring strong highs or deep lows might imply a rise in extreme weather events. Research suggests there has been an increase in precipitation intensity and floods in the UK since the 1960s (Osborn and Hulme, 2002; Fowler and Kilsby, 2003; Pattison and Lane, 2012; Jones *et al*., 2013a, 2014; Foulds and Macklin, 2016), so further work investigating these changes in relation to the changes in weather pattern frequencies may be valuable.

Changes in pattern frequencies are also comparable to changes in frequencies of LWTs derived from reanalysis products. Jones *et al*. (2013b) highlight the most pronounced LWT trends as a decline in easterly LWTs between 1871 and the 1920s, and an increase in frequency of north‐westerly LWTs between 1871 and 2010. Correspondingly, the only patterns in MO‐30 with easterly flow components (according to their LWT classification), WP27 and WP28, also decrease in frequency until the 1920s. The north‐westerly LWT changes are matched by a strong increase in the occurrence of WP13. The other north‐westerly variants, WP14 and WP26, show less suggestion of an upward trend, implying the increase in NW LWTs corresponds almost totally to the increase in WP13. In addition, Jones *et al*. (2013b) demonstrate that there is a slight increase in the frequency of westerly LWTs between 1871 and 2010. Of the four weather patterns in MO‐30 assigned as W LWTs, two show similar behaviour (WP10 and WP23), one shows the opposite (WP4) and one shows no change (WP20) over the same period. It is important to note that changes in observation density in the original MSLP products (i.e. the reanalysis data used in the MO‐30 and LWT derivations) may introduce artificial change points or trends in pattern frequencies. The EMULATE data set used for MO‐30 consists of land station and ship observations in the period 1850–1881, with data beyond 1881 blended with a pre‐existing data set. Prior to analysis, standard change point tests were performed on MO‐8 and MO‐30 frequencies, with no significant results found for this year. Therefore trends and change points reported in this article are unlikely to be due to changes in the observation network in the original MSLP data.

### 5.2 MO‐30 comparison with LWTs

The six most frequent LWTs in the period 1871 to 2015 are the pure anticyclonic and cyclonic types plus four westerly and southerly variants (Table 3). Of the patterns in MO‐30, three or four are mapped, via the objective method of Jenkinson and Collison (1977), to each of the six most frequent LWTs. Furthermore, the lack of easterly and north‐easterly LWTs in the set of 30 patterns is reflected by their relative rarity, with E, AE, CE, NE, ANE and CNE LWTs accounting for a combined occurrence of just 6.67%. It is perhaps surprising that more of the patterns in MO‐30 are not classified as pure anticyclonic LWTs, given the predominance of this type in the record (20.58%). Another interesting feature is that two patterns are assigned to the ‘unclassified’ LWT, which has a frequency of just 1.06%. Moreover, these two patterns occur relatively often (WP1 and WP7 account for a combined 11.37% of all pattern occurrences 1850–2015). We also counted MO‐30 pattern occurrences on the days of each LWT between 1871 and 2015 (Figure S4). The most frequent pattern for each LWT does not always correspond to that pattern's average LWT classification. Often, however, the pattern will feature similar behaviour to the more frequent patterns in terms of flow direction and cyclonicity. For example, on CW LWT days, WP30 is the fifth most frequent pattern, yet its MSLP definition as a LWT is CW. This is explained partly by the fact that pattern definitions are composites of individual MSLP days and partly by WP30 occurring less often than other patterns. As with WP30, the four most frequent patterns on CW LWT days (WP8, WP4, WP1 and WP26) all feature westerly flow, with WP8 and WP26 additionally being cyclonic (Figure 1).

The distinction MO‐30 makes between patterns that are defined as the same LWT is important. This distinction is linked to the larger region size used in the derivation of MO‐30 compared to LWT. The inclusion of much more of the North Atlantic Ocean and Europe allows for a wider view of the dominant large‐scale weather system for each pattern. For example, WP13 and WP14 are both defined as NW LWTs. Figure 1 shows that both patterns feature an anticyclone southwest of the UK and a cyclone to the northeast, causing a north‐westerly flow over the region. However, in WP13 the anticyclone is closer to the UK and the cyclone further away than for WP14, resulting in WP13 being drier than WP14 overall (Figure 6). WP26 is also classed as a NW LWT, with the low and high in similar positions to WP14. The depth of the low pressure anomaly is much greater however, and WP26 is the sixth wettest pattern over the UK. Subtleties between other groups of weather patterns with the same LWT assignment are also evident (e.g. WP2, WP12, WP15 and WP21, which are all SW LWTs). In total, 18 of the 30 patterns feature some kind of westerly flow over the UK, compared to the nine possible westerly LWTs (W, SW and NW plus the A‐ and C‐directional hybrids). By having a greater number of patterns representing the most common flow direction over the UK (i.e. westerly), more precise statements may be made about the precipitation expected. This could be useful in forecasting and in historical analyses inferring precipitation amounts from the weather pattern on that day.

### 5.3 Suitability of MO‐8 and MO‐30 in UK‐based precipitation analyses

To be useful in precipitation‐based analyses, precipitation distributions should be distinct between weather patterns. Section 3.1 demonstrates that this is the case for MO‐30, with most patterns exhibiting differences in median precipitation amount or variability. Furthermore, the distinction between patterns is generally greater than for LWTs, evidenced particularly by high variability in some of the wetter LWTs (Figure 7). MO‐8 was produced by clustering patterns from MO‐30 according to the spatial correlation of MSLP anomalies. This combines patterns from MO‐30 with very different precipitation distributions and hence the resulting pooled precipitation distributions for some groups of patterns in MO‐8 are very similar to each other. Therefore, although smaller sets of weather patterns may be preferable in some applications, we recommend that for UK precipitation a different clustering method should be used to group patterns from MO‐30. A weather pattern classification with this design specification might include some atmospheric water component in the derivation. For example, for the contiguous USA, Prein *et al*. (2016) classify weather patterns using sea level pressure, precipitable water and 700 hPa wind speed, as these variables are crucial in the physical processes driving precipitation (Doswell *et al*., 1996).

Results for monthly frequency anomalies of weather patterns during dry/wet periods, defined by SPI thresholds, demonstrate the comparability of MO‐30 and LWTs. In the original description of LWTs, Lamb (1972) describes anticyclonic and cyclonic types as dry and wet, respectively, across the UK, with regional differences in precipitation evident among the directional types. This general wet/dry behaviour mostly agrees with precipitation associated with MO‐30 patterns: westerly variants typically occur more (less) often than the mean during wet (dry) periods in western regions and less (more) often than the mean during wet (dry) periods in eastern regions. The opposite is true for easterly types.

### 5.4 Weather patterns associated with drought

In general, the weather patterns associated with droughts are physically consistent with expected conditions (in terms of airflow direction and cyclonicity). Patterns defined by flow from one direction tend to occur more often than normal during droughts in regions on the opposite side of the UK, and less often than normal during droughts in regions closer to the airflow direction source. Patterns characterized by anticyclonic behaviour over a region are more likely to enhance drought and vice versa. This is in agreement with Phillips and McGregor (1998), who showed that several droughts in southwest England between 1962 and 1996 were mostly characterized by an increase in the occurrence of the N, NE, E and SE LWTs and their anticyclonic equivalents. This was coincident with a decrease in southerly and westerly LWTs. However, it is interesting to note that the pattern showing the largest departure in frequency during six‐month droughts in SWE is WP23 (Figure 13). This patterns' LWT equivalent is W and so intuitively wet conditions would be expected, yet from the MSLP definition shown in Figure 1, SWE is close to the centre of high pressure and would therefore experience calmer, drier conditions than other regions. In Yorkshire (part of NEE), Fowler and Kilsby (2002b) found that droughts were associated with increases in the A, AE, ASE, AS and ASW LWTs, sometimes with concurrent decreases in westerly LWTs. Correspondingly, DSI‐3 droughts in NEE coincide with increases in the frequency of several patterns from the new classification system, such as WP3 (ASW), WP6 (A), WP12 (ASW), WP16 (AS), WP17 (ASE) and WP18 (ASW). There is also some agreement for weather patterns that decrease in occurrence during droughts. Notable exceptions are WP19 (N), WP24 (CN) and WP28 (CSE), which are in the “easterly” cluster in Fowler and Kilsby (2002b), and WP27 (AE). Disagreement between MO‐30 and LWT frequencies such as this highlights how pattern definitions in MO‐30 are more subtle, and direct analogy to a particular LWT may be unsuitable. In general, there are difficulties in comparing different classifications, as often they have different numbers of weather patterns, are calculated for different domains and use different input data sets.

## 6 Conclusions

This study demonstrates the applicability of a new set of 30 weather patterns to UK precipitation and meteorological drought analyses, and its advantages compared to LWTs. Weather patterns in this classification mostly show more distinct differences in the daily precipitation distribution for nine UK regions than is the case for LWTs. A smaller set of eight patterns, however, as currently defined is not suitable for UK rainfall and meteorological drought analysis as weather patterns show too much similarity in their precipitation amounts. Monthly frequency anomalies show which patterns occur more or less frequently during SPI‐defined dry or wet periods across all regions, and any regional differences. It is demonstrated that, in general, the same patterns are responsible for dry or wet periods for time‐scales of 3, 6 and 12 months. The magnitude of anomalies associated with these patterns typically decreases as the time‐scale is increased. Patterns associated with dry (wet) conditions typically occur more (less) often than normal during drought months, which are defined using the DSI. Regional differences in the weather patterns associated with SPI dry conditions mostly hold for the DSI equivalent. Weather patterns associated with drought can be summarized as follows.

- During droughts spanning the majority of UK regions, WP6, WP9, WP10, WP12, WP17 and WP25 occur more often than normal.
- Droughts in western (eastern) regions are generally accompanied by a rise (fall) in the frequency of WP27.
- Droughts in eastern (western) regions often see increases (decreases) in the number of WP15 days.
- WP23 occurs more often than normal during droughts in all regions except those in Scotland.

There are several opportunities for further research. Clustering weather patterns from MO‐30 into a smaller set based on precipitation, in addition to spatial correlation of MSLP anomalies, might be useful for monthly or seasonal analyses where fewer patterns are desired. Furthermore, the methods presented in this article could be applied to other regions in Europe, to worldwide regions using another weather pattern data set, and to hydrological drought with a different drought index. Finally, the predictability of MO‐30 can be investigated, with a focus on the high‐risk drought patterns highlighted in this article. As results here consider weather pattern frequency anomalies on time‐scales of at least 3 months, any forecast product arising from this research would require a minimum 3‐month lead‐time. There are several global seasonal forecast models capable of providing probabilistic forecast output to at least this range (MacLachlan *et al*., 2015), enabling a prediction of the spread of forecast frequency anomalies. This could form the basis for a probabilistic forecaster decision tool, highlighting periods with a higher likelihood of drought conditions months in advance.

## Supporting information


**Figure S1.** As Figure 8, but for wet periods defined by SPI‐3 ≥1.
**Figure S2.** As Figure 8, but for summer, wet periods defined by SPI‐3 ≥1.
**Figure S3.** As Figure 8, but for winter, wet periods defined by SPI‐3 ≥1.
**Figure S4.** Percentage occurrence of each weather pattern in MO‐30 for each LWT day between 1871 and 2015. Rows sum to 100%.
