# Supplementary material for: A new precipitation and drought climatology based on weather patterns
Source: Int J Climatol. 2017 Jul 13;38(2):630–48. doi: 10.1002/joc.5199 (PMC5812058; doi:10.1002/joc.5199)
Supplement: Supplementary file 1 — Figure S1. As Figure 8, but for wet periods defined by SPI‐3 ≥1. Figure S2. As Figure 8, but for summer, wet periods defined by SPI‐3 ≥1. Figure S3. As Figure 8, but for winter, wet periods defined by SPI‐3 ≥1. Figure S4. Percentage occurrence of each weather pattern in MO‐30 for each LWT day between 1871 and 2015. Rows sum to 100%. [file JOC-38-630-s001.docx]

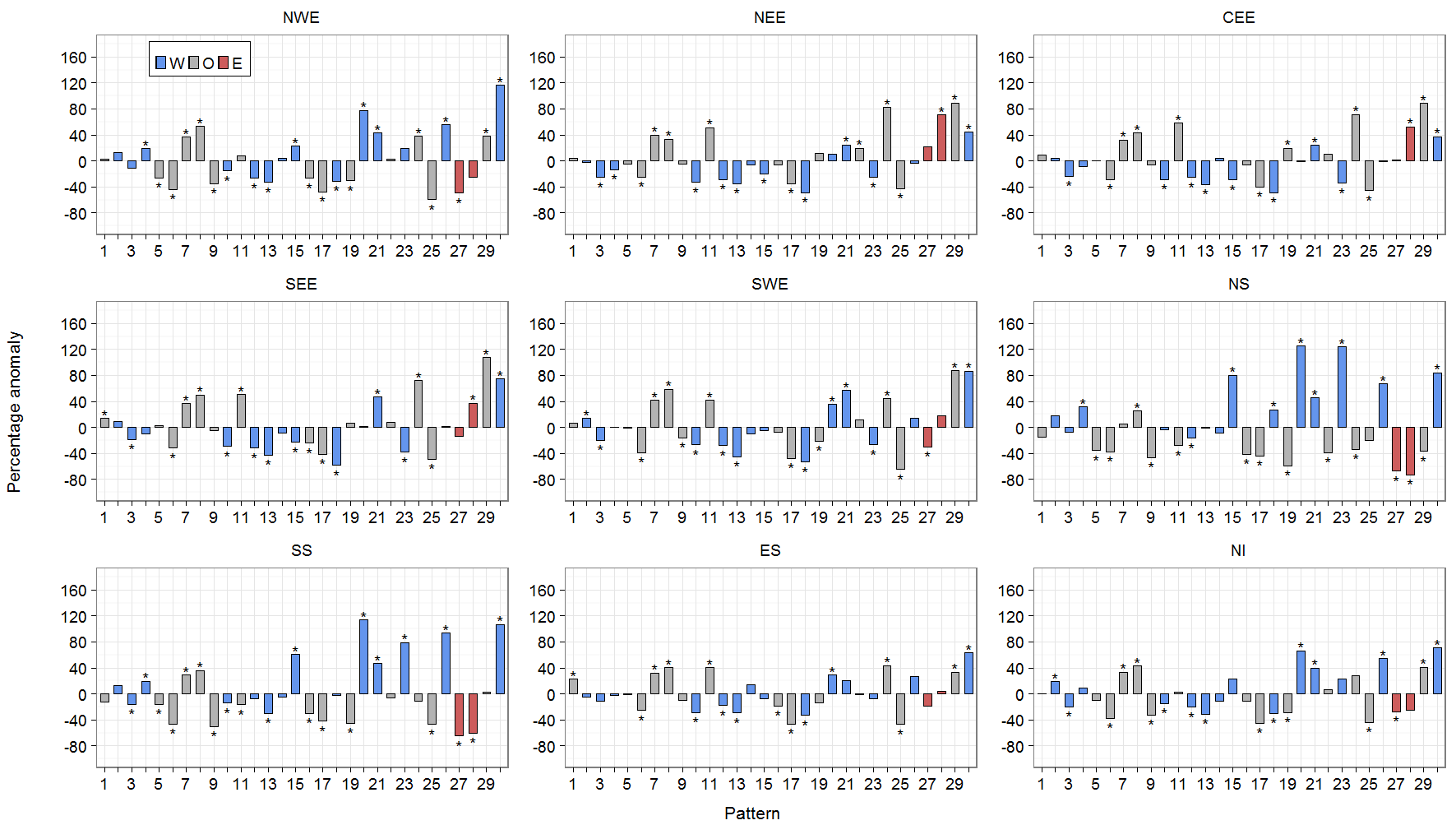
Figure S1: As Figure 8, but for wet periods defined by SPI-3 $\geq1$.
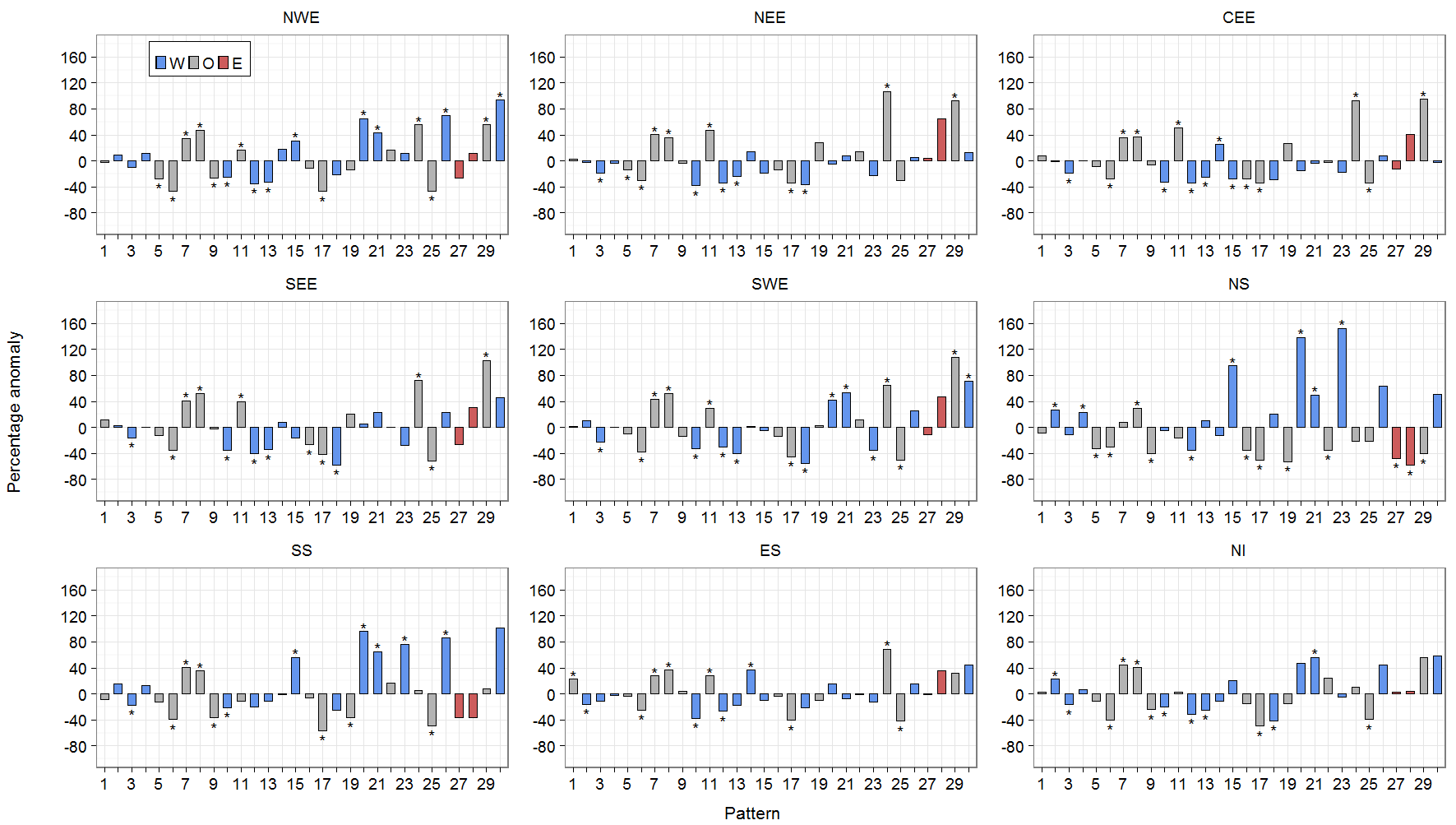


Figure S2: As Figure 8, but for summer, wet periods defined by SPI-3 $\geq1$.


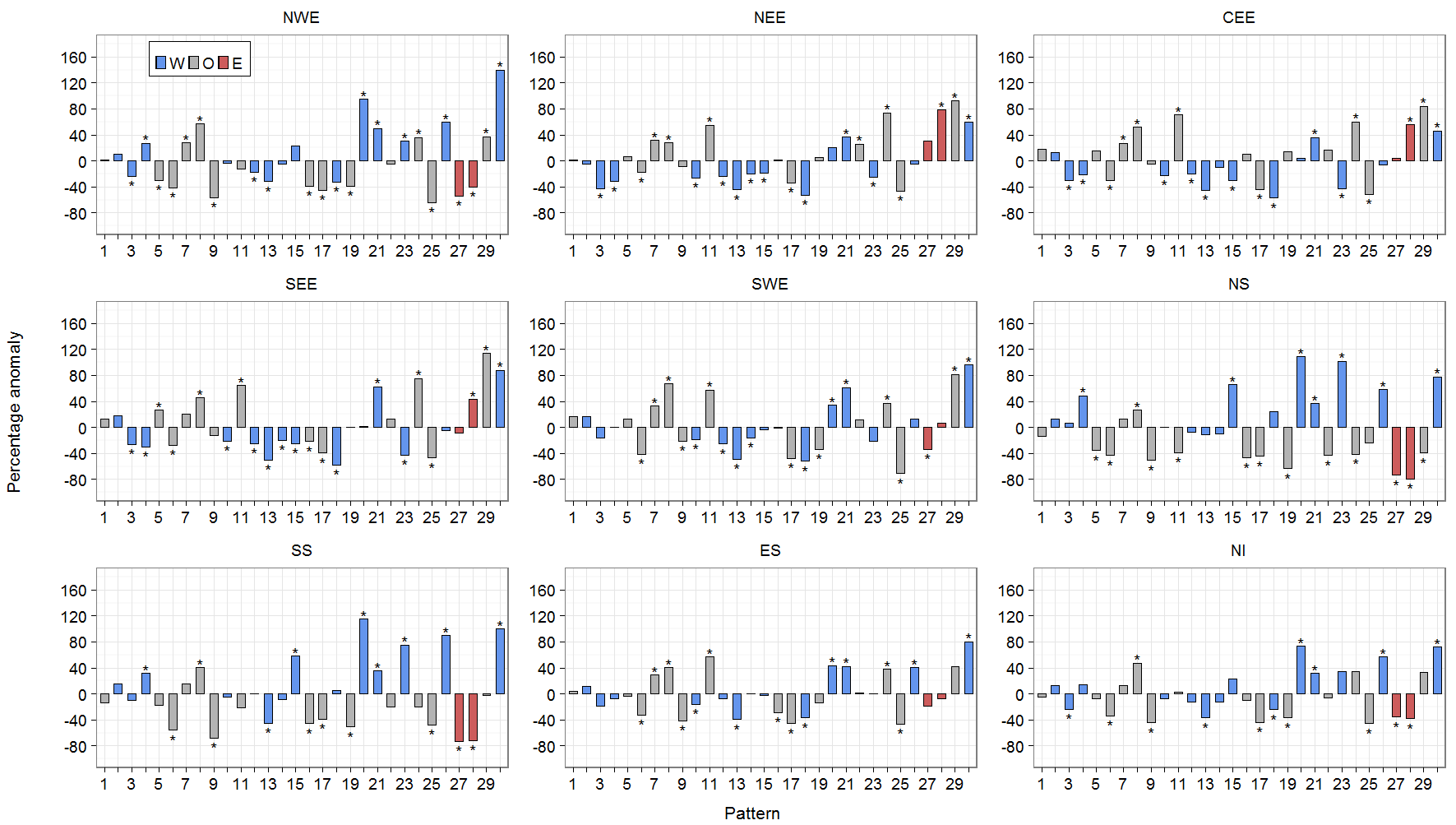


Figure S3: As Figure 8, but for winter, wet periods defined by SPI-3 $\geq1$.


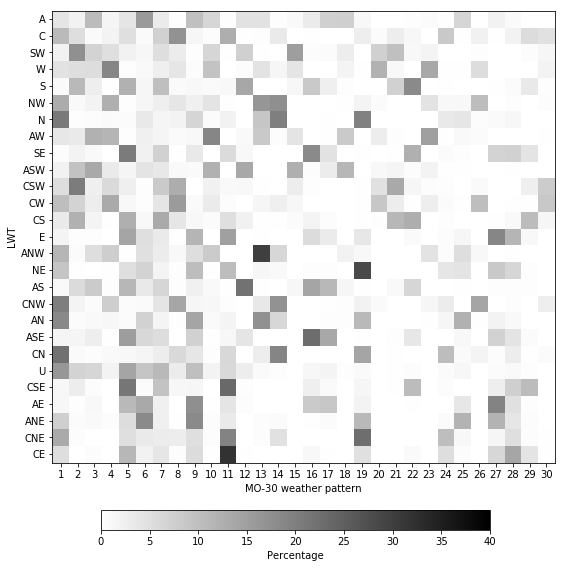


Figure S4: Percentage occurrence of each weather pattern in MO-30 for each LWT day between 1871 and 2015. Rows sum to 100%.
